# Direct Ink Writing of Phenylethynyl End-Capped Oligoimide/SiO_2_ to Additively Manufacture High-Performance Thermosetting Polyimide Composites

**DOI:** 10.3390/polym14132669

**Published:** 2022-06-30

**Authors:** Keda Li, Jinghong Ding, Yuxiong Guo, Hongchao Wu, Wenwen Wang, Jiaqi Ji, Qi Pei, Chenliang Gong, Zhongying Ji, Xiaolong Wang

**Affiliations:** 1 State Key Laboratory of Applied Organic Chemistry, Key Laboratory of Special Function Materials and Structure Design of Ministry of Education, College of Chemistry and Chemical Engineering, Lanzhou University, Lanzhou 730000, China; likd21@lzu.edu.cn (K.L.); dingjh18@lzu.edu.cn (J.D.); wuhc19@lzu.edu.cn (H.W.); wangww20@lzu.edu.cn (W.W.); jijq21@lzu.edu.cn (J.J.); peiq20@lzu.edu.cn (Q.P.); 2 State Key Laboratory of Solid Lubrication, Lanzhou Institute of Chemical Physics, Chinese Academy of Sciences, Lanzhou 730000, China; guoyuxiong91@163.com; 3 Shandong Laboratory of Yantai Advanced Materials and Green Manufacturing, Yantai Zhongke Research Institute of Advanced Materials and Green Chemical Engineering, Yantai 264006, China

**Keywords:** direct ink writing, thermosetting polyimide, dimensional shrinkage, thermal stability

## Abstract

The three-dimensional (3D) printing of a SiO_2_-filled thermosetting polyimide (SiO_2_@TSPI) composite with outstanding performance is realized via the direct ink writing (DIW) of polyamide acid (PAA) composite ink and thermal treatment conducted thereafter. The composite ink consists of phenylethynyl-terminated PAA and silica nanoparticles, where the SiO_2_ nanoparticles serve as the rheology modifier that is necessary for the DIW technique to obtain self-supporting feedstock during 3D printing and the reinforcement filler that is used to enhance the performance of the final composite. As a result, printed parts with complex geometry and robust thermal stability are obtained. Due to the extrusion-based DIW technique, the printed structures exhibit anisotropic mechanical strength that highly depends on printing roads. This simple and convenient means of realizing 3D structures of thermosetting polyimides is a promising strategy in aerospace and other fields.

## 1. Introduction

Polyimides (PIs) as a class of high-performance engineering materials have drawn considerable attention in tribology, aerospace, separating membranes, and micro-electronic applications owing to their diversity of synthetic routes and impressive properties, such as excellent mechanical strength, high long-term operating temperature, low dielectric constant, and chemical and radiation resistance [1,2,3,4]. Among the various polyimides, the thermosetting polyimide (TSPI) has attracted increasing attention due to its high glass transition temperature (Tg) and thermal stability, as it can be used above 300 °C for a long time. To date, plenty of thermosetting polyimides with diverse curing groups, including maleimide, norbornenimide, benzocyclobutene, cyano, and alkynyl groups, have been developed [5,6,7,8,9], all of which exhibit excellent thermo-oxidative stability, aging resistance, and outstanding toughness, and, therefore, extensive potential. However, the melting temperature of thermosetting polyimides cannot be observed, and their poor solubility makes the fabrication process difficult, which impedes their applications, especially when architectures with high complexity are necessary. In general, traditional compression molding and sintering with the assistance of high pressure or high temperature are used to manufacture architectures, but they need both expensive molds and complicated processes that are time- and cost-consuming [10]. In addition, the mass fabrication of molds and machining will inevitably bring about a waste of resources.

Fortunately, the emergence of additive manufacturing (AM) provides the option of addressing the above challenges. AM, usually referred to as three-dimensional (3D) printing, has shown great promise in translating digital manufacturing into high-performance components, with the merits of fabricating complex geometric structures and minimizing the waste of materials, and it has been widely employed in the fields of engineering, soft robots, sensors, energy, and so on [11,12,13,14,15]. To date, several 3D printing techniques have been developed, including digital light processing (DLP), direct ink writing (DIW), fused deposition modeling (FDM), selective laser sintering (SLS), and stereolithography apparatus (SLA) [16,17,18,19,20], and these have been employed to achieve the AM of a variety of materials, such as polyetheretherketone [21], polydimethylsiloxane (PDMS) [22], epoxy resin (ER) [23], cyanate ester (CE) [24], and ceramic [25,26]. As one of the most important materials in modern life, the AM of PI is very promising. For instance, Hegde et al. [27] employed mask projection stereolithography (MPSL) to fabricate all-aromatic 3D printed polyimide structures with outstanding thermal properties and a complicated geometric shape. Rau et al. [28] used UV-assisted DIW to manufacture all-aromatic polyimide parts with excellent thermal performance. Guo et al. [29] and Li et al. [30] adopted the approach of grafting photoactivity groups to prepare polyimide parts and even shape memory polyimide objects using DLP technology. Despite the 3D architectures of the above techniques displaying excellent performance and complex geometry, large dimensional shrinkage occurred, and only a few of the architectures could retain high strength and good thermal properties simultaneously. Therefore, the 3D printing of polyimides with excellent performance and complicated geometric shapes remains a challenge.

Direct ink writing (DIW) possesses a mild temperature during the printing process and employs self-supporting viscoelastic materials as feedstock. Thus, after the self-supporting inks are deposited onto substrates, they maintain their geometry as designed. In this work, based on the purpose of utilizing the DIW 3D printing technique to obtain robust 3D printed composite objects via the optimization of formulation and post curing, the ink consisted of an oligomer with a structure of phenylethynyl-terminated polyamic acid and the silica nanoparticles that served as both reinforcement fillers and rheology modifiers. The strength of the printed filaments and the adhesive force among the layers were evaluated. As a result, the printed routes of specimens were divided into transverse (X) and axial (Y) orientations. The microstructure, the shrinkage value before and after thermal treatment, the thermal performance, and the mechanical properties’ dependence on printing routes for printed parts were also investigated.

## 2. Materials and Methods

### 2.1. Materials

4,4′-(hexafluoroisopropylidene) diphthalic anhydride (6FDA), 4-phenylethynylphthalic anhydride (PEPA) and 4,4′-diaminodiphenyl ether (ODA) were purchased from HWRK CHEM Co., Beijing, China. Isobutanol was received from Zhongqin Chemical Reagent Co., Shanghai, China. 4,4′-diaminodiphenyl ether was recrystallized from isobutanol. 4,4′-(hexafluoroisopropylidene) diphthalic anhydride was dried at 120 °C under vacuum for 24 h. Calcium hydride was obtained from Damao Chemical Regent Co., Tianjing, China. Silica nanoparticles with a size of 15 nm were purchased from Ai Purui Nano Regent Co., Nanjing, China. *N, N*-dimethylformamide and *N*-methyl pyrrolidone were bought from Ke Miou Chemical Reagent Co., Tianjing, China. *N*-methyl pyrrolidone (NMP) was dried with calcium hydride for 12 h, was collected by reducing pressure distillation, and was stored over dried molecular sieves (4 Å). Other reagents were used as received.

### 2.2. Preparation of Thermosetting Polyimide and Polyamide Acid (PAA) Powders

**Scheme 1:**
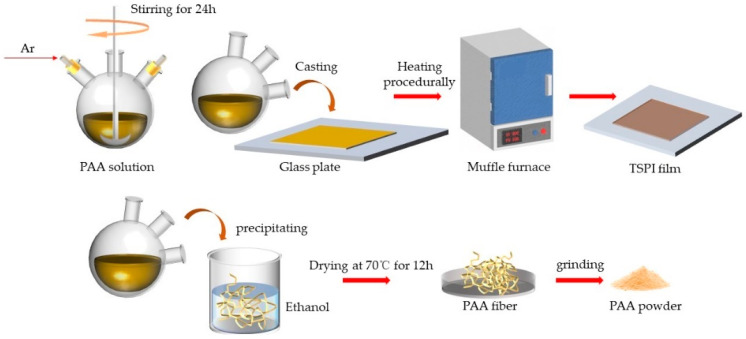
Preparation process of thermosetting polyimide film and polyimide acid (PAA powders).

The phenylethynyl-terminated PAA solution was synthesized according to the published literature [31]. ODA (24.57 mmol), 6FDA (23.57 mmol), and PEPA (2 mmol) were added to a 250 mL three-necked flask, which was equipped with a mechanical agitation system and an argon inlet/outlet. The total preparation procedure of the phenylethynyl-terminated polyamide acid (PAA) solution is shown in Scheme 1. First, after ODA was completely dissolved in NMP, the solution was moved into an ice bath for 20 min. Then, 6FDA was added gradually, and the reaction was stirred for 1 h in the ice bath. After that, the ice bath was removed, and the mixture was stirred for 5 h at room temperature. Then, the PEPA and the remainder of the NMP were added, the solution was stirred for another 24 h, and a phenylethynyl-terminated PAA solution with a solid content of 15% was obtained. The PAA solution was added to 300 mL of ethanol to obtain a PAA fiber. Then, the PAA fiber was filtered and dried at 70 °C for 12 h and smashed into powder. The cured thermosetting polyimide (C-TSPI) films were prepared by coating the PAA solution onto glass for thermal imidization and for the formation of a crosslink network in a muffle furnace in air environment, and the heat treatment route was as follows: 80 °C for 2 h, 120 °C for 1 h, 150 °C for 1 h, 180 °C for 1 h, 250 °C for 1 h, 280 °C for 2 h, 320 °C for 1 h, and 370 °C for 1 h. The uncured PI samples were prepared using the same procedure in the same conditions, but the heat treatment route was ended after running it at 280 °C for 2 h, both of them with a heating rate of 2 °C /min. M_n_ = 18,700 g/mol, PD = 1.38.

### 2.3. Preparation of Inks and 3D Printing of Thermoset Objects

**Scheme 2:**
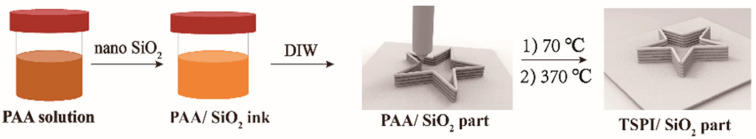
The process of preparation of 3D printed parts of SiO2@TSPI composite.

The used viscous inks consisted of PAA powders with structures of phenylethynyl-terminated, *N,N*-dimethylformamide (DMF), and SiO_2_ nanoparticles, which serve as matrix, solvent, and reinforcement fillers and rheology modifiers, respectively. The phenylethynyl-terminated PAA powders were dissolved in DMF using the ball-milling method, and a PAA solution with a solid content of 70% was obtained. A series of PAA composite inks with SiO_2_ weight contents of 6.2%, 16.3%, and 26.3% were realized by gradually adding different dosages of SiO_2_ nanoparticles into 70% PAA solution and dispersed uniformly by repeatedly using the ball-milling method. After that, the rheological behaviors of the composite inks were evaluated using a rheometer (HAAKE, RS6000, Germany), which was equipped with a parallel plate model with a 35 mm diameter, and their heights were fixed at 1 mm. The shear viscosity of the inks was measured at 25 °C with shear rates from 10^−2^ to 102 s^−1^. The storage modulus (G’) and loss modulus (G’’) were tested under room temperature at shear stress ranging from 0.1 to 6000 Pa with a frequency of 1 Hz. Viscoelastic inks were transferred into a syringe and degassed to remove air bubbles by centrifugation under the speed of 7000 r/min for 10 min before use. The inks with high viscosity were successfully extruded under the condition of additionally providing pneumatic pressure by shearing of the screw. The whole printing process is shown in Scheme 2. The syringe filled with inks was equipped with a gauge nozzle with a gauge of 18, 21, or 24 and an inner diameter of about 800 μm, 700 μm, or 300 μm, respectively. The process of thermal curing was conducted in a muffle furnace under an air atmosphere, and the whole curing procedure with a heating rate of 1 ^o^C /min was carried out as follows: 80 °C for 4 h, 120 °C for 4 h, 150 °C for 4 h, 180 °C for 2 h, 250 °C for 2 h, 280 °C for 2 h, 320 °C for 2 h, and 370 °C for 1 h. Casting SiO_2_@TSPI (C- SiO_2_@TSPI) composite films were thermally cured the same way.

### 2.4. Characterizations

Thermal stability and thermo-oxidative stability were measured using TA instruments (Netzsch STA 449C, Germany), and the tests were operated at a heating rate of 10 °C/min with the temperature from 25 °C to 800 °C under air and a nitrogen atmosphere, respectively. An electronic universal testing machine (SHIMADZU, EZ-Test, China) was used to evaluate the mechanical performances of cured PI and printed coupons at a load capacity of 500 N and at a speed of 5 mm/min. At least three independent test samples were used for each measurement. Pictures of the size, surface, and morphologies of objects and elemental analysis results were gained through optical microscopy (Olympus DP72, Japan) and scanning electron microscopy (SEM, JSM-6701, Japan) at 10 to15 KV. Gel permeation chromatography (GPC, Waters 1525) was utilized to calculate the molecular weight of the polyimide. The mobile phase was DMF, and styrene served as a standard sample. The vernier caliper was utilized to measure and calculate the volume of grids or cylinders before and after thermal treatment. Dimensional shrinkage was calculated as follows:

Dimensional shrinkage = (L1 − L2)/L1 × 100%

Volume shrinkage = (V1 − V2)/V1 × 100%

In the formula, the parameters of L1 (V1) and L2 (V2) are the dimension (volume) of constructs before and after heat treatment, respectively. The shrinkages were measured three times for every sample.

## 3. Results and Discussion

### 3.1. Chemical Structures of Uncured Polyimide and Cured Thermosetting Polyimide

**Figure 1:**
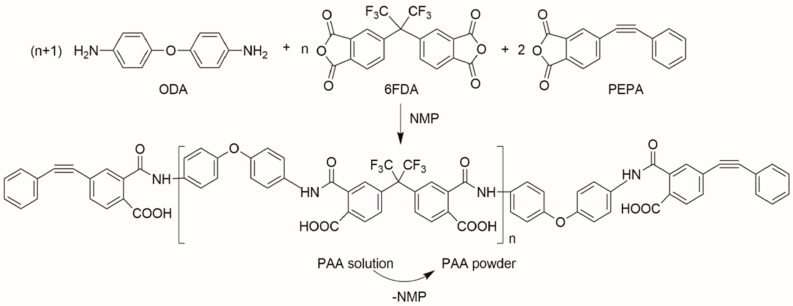
Schematic of the preparation of PAA powder.

**Figure 2:**
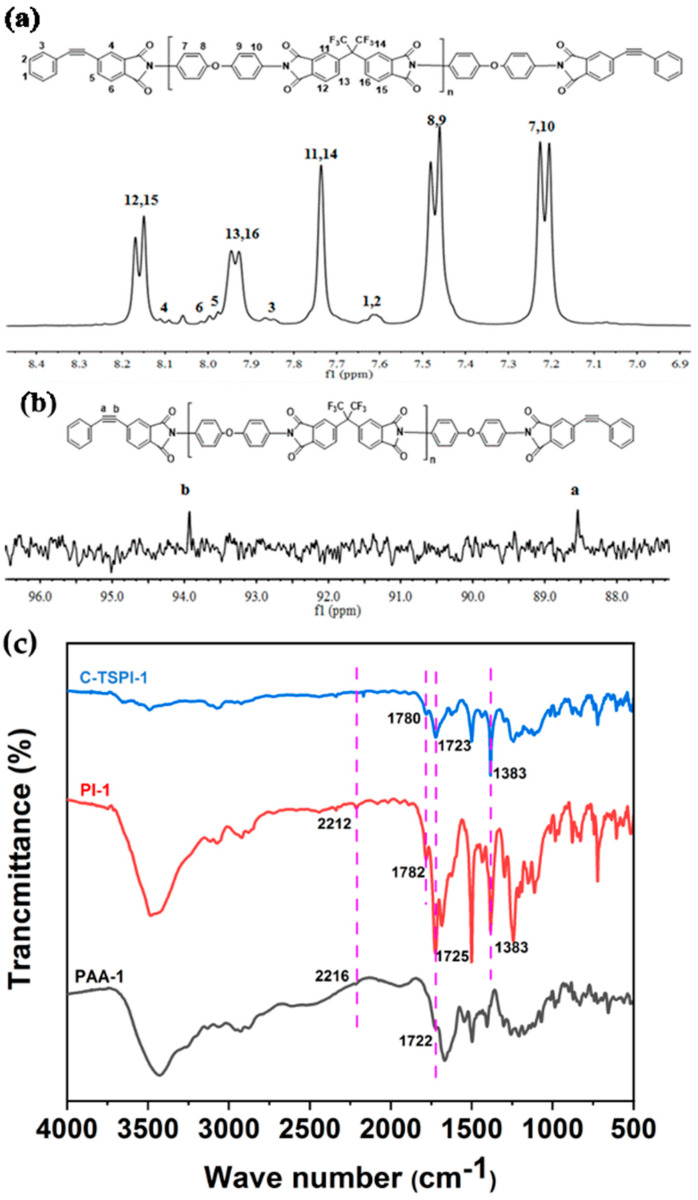
(**a**) ^1^H-NMR and (**b**) ^13^C-NMR spectra of uncured PI-1; (**c**) the FT-IR of PAA-1 powders, PI-1, and cured PI-1.

The process of synthesizing phenylethynyl-terminated polyimide by condensation of ODA, 6FDA, and PEPA is shown in Figure 1. Two kinds of polyimide with different molecular weights were designed, and detailed information is shown in Table 1. The PI-1 oligoimide with a designed molecular weight of 5000 g/mol was synthesized in order to clearly investigate the crosslinking of phenylethynyl in the polyimide structure. The structures of uncured PI-1 and cured PI-1 were characterized using Fourier transform infrared spectroscopy (FT-IR) and nuclear magnetic resonance (NMR) (Figure 2). Figure 2a shows the ^1^H NMR spectrum of the uncured PI-1 oligomer, and all of the protons in the chemical structure of PI-1 are assigned as expected, implying that the obtained oligoimides have the ideal chemical structures. For the uncured PI-1 oligomer, the weak resonances at 88.54 ppm and 93.93 ppm could be assigned to the existence of the C≡C group (Figure 2b). Furthermore, Figure 2c compares the FTIR spectra of the PEPA oligoimides, uncured PI and cured PI. The absorption peak at 2216 cm^−1^ demonstrates the stretching vibrations of ethynyl groups (—C≡C—) in the oligoimides. The absorption peaks at 1779 and 1721 cm^−1^ are assigned to the vibration of the C=O asymmetric and symmetric stretching absorption bands, respectively. Meanwhile, the absorption peak at 1383 cm^−1^ belongs to the stretching vibration of C-N of the imine five-membered ring. These results suggest that phenylethynyl-terminated PI was successfully prepared via heat imidization. Compared to the C≡C peak of oligomer uncured PI-1, that at 2212 cm^−1^ disappeared after curing at 370 °C for 1 h, causing C≡C to react and thereby form a crosslink network in the heat treatment process [32].

### 3.2. Three-Dimensional Printing of SiO_2_@TSPI Composites Using Direct Ink Writing (DIW)

**Figure 3:**
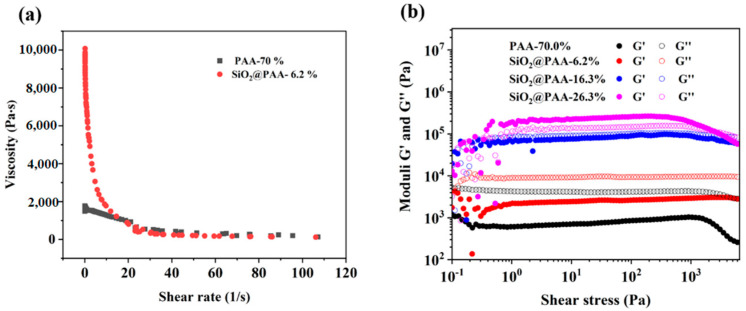
(**a**) Viscosity as a function of shear rate; (**b**) storage moduli and loss moduli as a function of shear stress.

Proper rheology is vital for DIW technology, and a trade-off between viscosity and high elastic modulus is required to keep the shape during and after extrusion. The rheological properties of the SiO_2_/PAA inks were analyzed using a rheometer. The PAA powder was dissolved in DMF to obtain a solution with a concentration of 70 wt%. As shown in Figure 3a, when SiO_2_ nanoparticles were added into the PAA solution, the viscosity of the mixture significantly increased compared with the PAA solution and exhibited the characteristic of non-Newtonian fluid. Both the inks of PAA-70% and SiO_2_@PAA-6.2% showed shear thinning behavior. Besides viscosity, the moduli test is another prerequisite for DIW ink. Figure 3b shows the storage modulus (G′) and loss modulus (G″) under different shear stresses. We can see that the storage modulus is lower than the loss modulus for PAA-70% and SiO_2_@PAA-6.2%. For SiO_2_@PAA-16.3%, the G′ is almost same as the G″. Further increasing the addition of SiO_2_ to 26.3% makes the G′ higher than the G″, which means that only SiO_2_@PAA-26.3% exhibits the phenomenon of self-support (Figure 3b). The storage modulus overtakes the loss modulus at shear stress below 581 Pa. Therefore, SiO_2_@PAA-26.3% was chosen to print a variety of structures in order to investigate numerous properties. 

**Figure 4:**
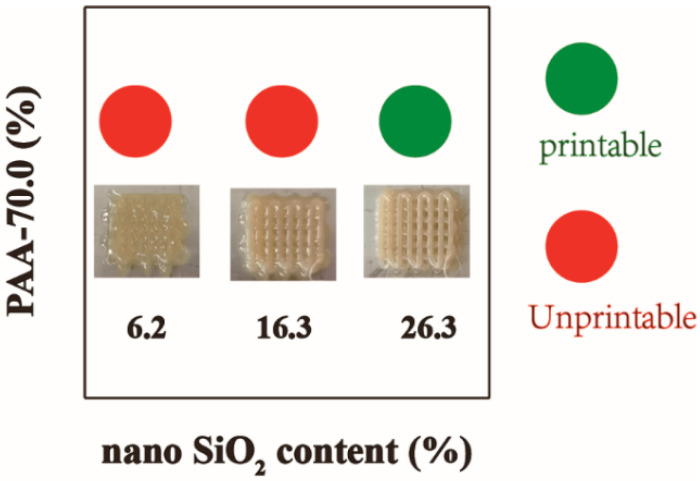
The printability of SiO_2_@PAA composite inks.

We then studied the printability of the above inks. The inks of SiO_2_@PAA-6.2% and SiO_2_@PAA-16.3% could extrude filaments, and they could deposit onto glass to form grids. However, after a few minutes, the grids had collapsed (Figure 4). The grids originating from SiO_2_@PAA-26.3% had maintained their shape before and after heat treatment and showed good dimensional stability. Then, the parameters for SiO_2_@PAA-26.3% ink were adopted to SiO_2_@PAA inks.

**Figure 5:**
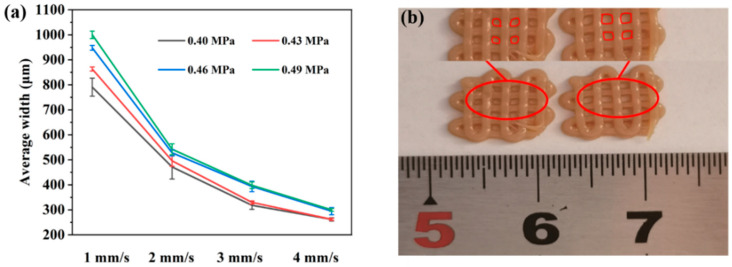
The printable parameters of ink: (**a**) the widths of the single filaments when the speed of the nozzle was 1–4 mm/s and the extrusion pressure changed from 0.40 MPa to 0.49 MPa; (**b**) the pull interaction between the filaments (the z-spacing of the two left and two right parameters are 0.4 mm and 0.6 mm, respectively).

As the resolution of 3D printed objects is primarily affected by the extrusion pressure, the z-spacing (the distance of the nozzle to the substrate), and the moving speed of the nozzles, we investigated the width of a single filament under different extrusion pressures and moving speeds of the nozzles to achieve suitable conditions for DIW (Figure 5a). The width of a single filament was measured by fixing the shear speed of the screw and by changing the extrusive pressure, z-spacing, and moving speed of the nozzles. The single filaments were deposited onto substrates, and their widths were measured. The filaments were obtained under the condition of extrusion pressures between 0.40 to 0.49 MPa with the moving speed of the nozzle varying from 1 to 4 mm/s. The higher extrusive pressures for the inks and the lower moving speeds of the nozzle corresponded to the more extrusive inks, which produced larger widths for every filament. An appropriate z-spacing is vital for printing geometry parts to avoid previously printed dragging filaments. As presented in one such instance (Figure 5b), the extrusion pressure, the moving speed of the nozzle, and the shear speed of the screw were kept constant, while the z-spacing was varied to fabricate neat grids. After completing the above investigation, the parameters to print random structures using SiO_2_@PAA-26.3% ink were determined, because they are composed of single filaments.

**Figure 6:**
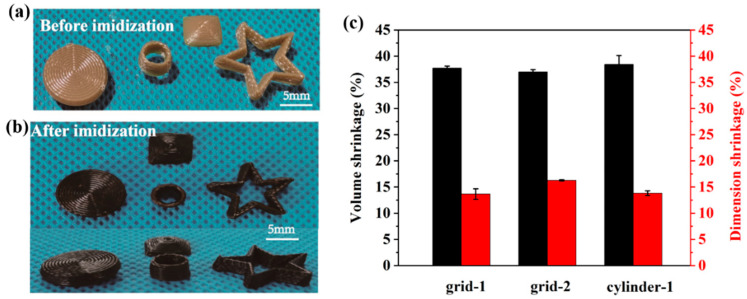
(**a**,**b**) Examples of printed structures before and after heat treatment; (**c**) the volume and dimensional shrinkage of the printed structures.

In order to avoid the formation of cracks, guarantee shape stability, and fully release the internal stress during the heat treatment process for uncured parts, different curing procedures were utilized to prepare C-TSPI and C-SiO_2_@TSPI films and printed parts for SiO_2_@TSPI-26.3%. As the printed materials are thicker than films, resulting in different solvent volatilization rates between the surface, inner structures, and the larger internal stress, the printed materials need more time and a lower heating rate to decrease the influence by weakening the releasing rate of inner stress during the process of thermal treatment. The dimensional shrinkage of the structures was measured, and the average values were in the range of 13.65% to 16.27% (Figure 6).

**Figure 7:**
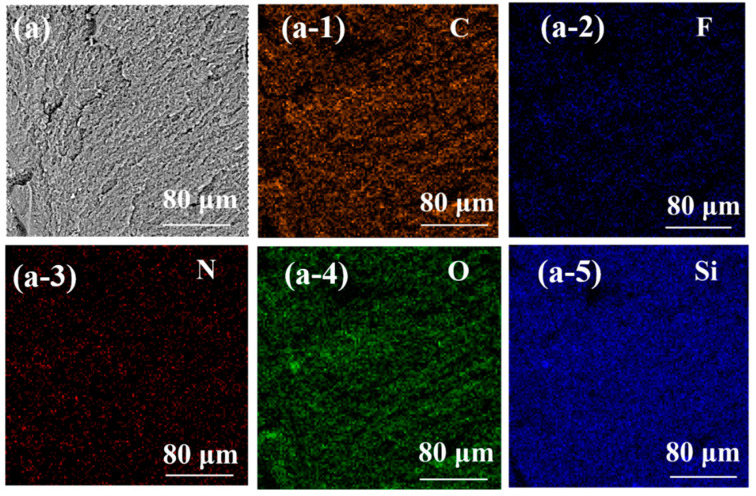
(**a**) The SEM microstructure for the cross-section of the printed SiO_2_@TSPI composite film; (**a-1**)–(**a-5**) the EDS results for the printed SiO_2_@TSPI composite.

**Figure 8:**
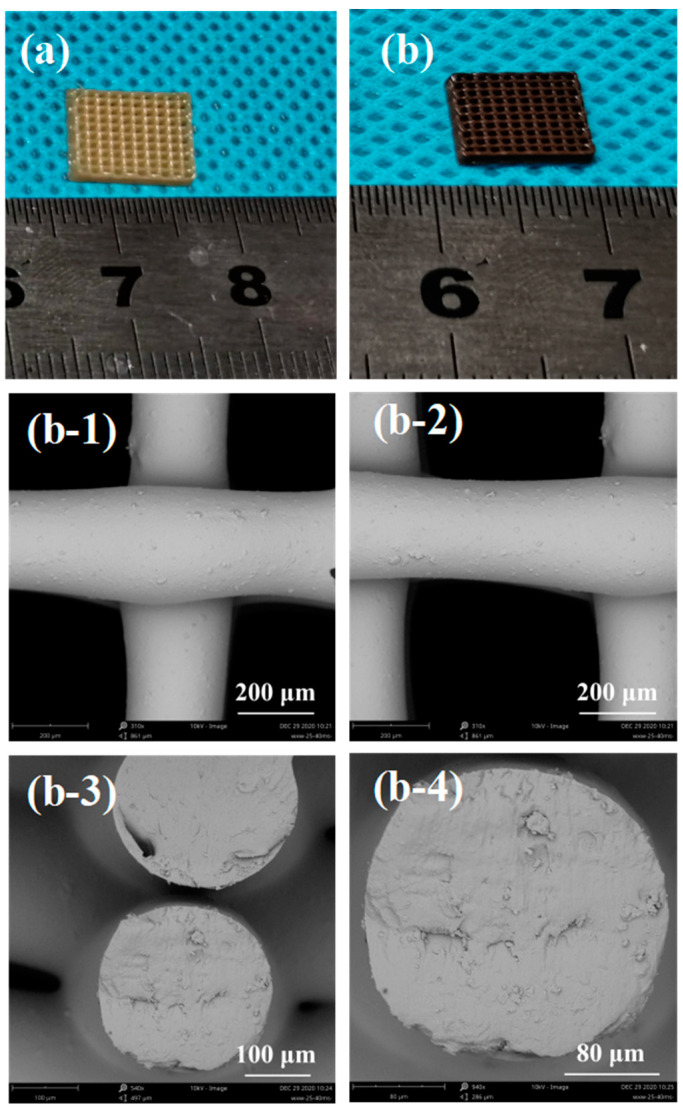
Images of the (**a**) uncured and (**b**) cured printed SiO_2_@TSPI composites; (**b-1**) and (**b-2**) SEM images of the cured printed SiO_2_@TSPI scaffold section; (**b-3**) and (**b-4**) SEM images of the cured printed SiO_2_@TSPI cross-section.

The surface morphology of the printed SiO_2_@TSPI-26.3% material was analyzed using SEM. In Figure 7, from the EDS of Si, it can be seen that the distribution of nano-SiO_2_ was uniform. The results suggest that the nano-SiO_2_ particles were well-scattered in the oligomer ink. In Figure 8a,b, it can be observed that the printed grid maintained its shape after being cured. The microstructure of the printed grid was also observed. The results show that the filaments were separately positioned stack by stack and kept their shape, which indicates the outstanding self-supporting properties of the composite ink.

### 3.3. Thermal Stability of TSPI Films and Printed SiO_2_@TSPI-26.3%

**Figure 9:**
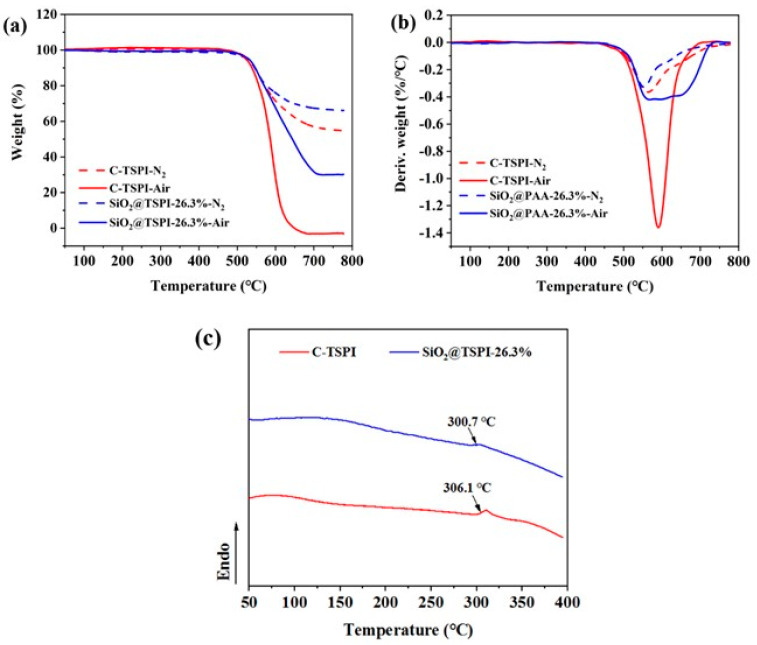
(**a**,**b**) Thermal performance of TSPI and printed SiO_2_@TSPI composites in air and nitrogen atmosphere; (**c**) DSC curves of TSPI and printed SiO_2_@TSPI composites.

The TGA and DSC of the C-TSPI films and printed SiO_2_@TSPI-26.3% were measured in air and nitrogen as shown in Figure 9 and Table 2. The T_g_ of both the C-TSPI film and printed SiO_2_@TSPI-26.3% was above 300 °C. The TGA results indicate that all the samples exhibited outstanding thermal properties and thermo-oxidative stability. There was no significant decline in the weight of the C-TSPI film or the printed SiO_2_@TSPI-26.3%, even under 500 °C in air and a nitrogen atmosphere. The degradative peak for C-TSPI was mainly caused by the degradation of the polymer from 552.0 °C to 612.0 °C, while the destructive peak for the printed SiO_2_@TSPI-26.3% occurred due to the degradation of the polymer from 554.0 °C to 714.0 °C. For SiO_2_@TSPI-26.3%, the destructive peak with a lower decomposition rate and a broader decomposition temperature suggests a composite with enhanced thermo-oxidative stability. At the same time, the char yield at 770 °C of C-TSPI in the nitrogen atmosphere was 54.71%, while the char yield for the printed SiO_2_@TSPI-26.3% was up to 65.96%. The above results indicate that the composite’s higher thermal properties and thermo-oxidative stability are attributed to the nano-SiO_2_ with outstanding thermal performance. The incorporation of nano-SiO_2_ contributed to the T5%, and the char yields of the composites increased under air and the nitrogen atmosphere.

### 3.4. Mechanical Properties of Printed Specimens

**Figure 10:**
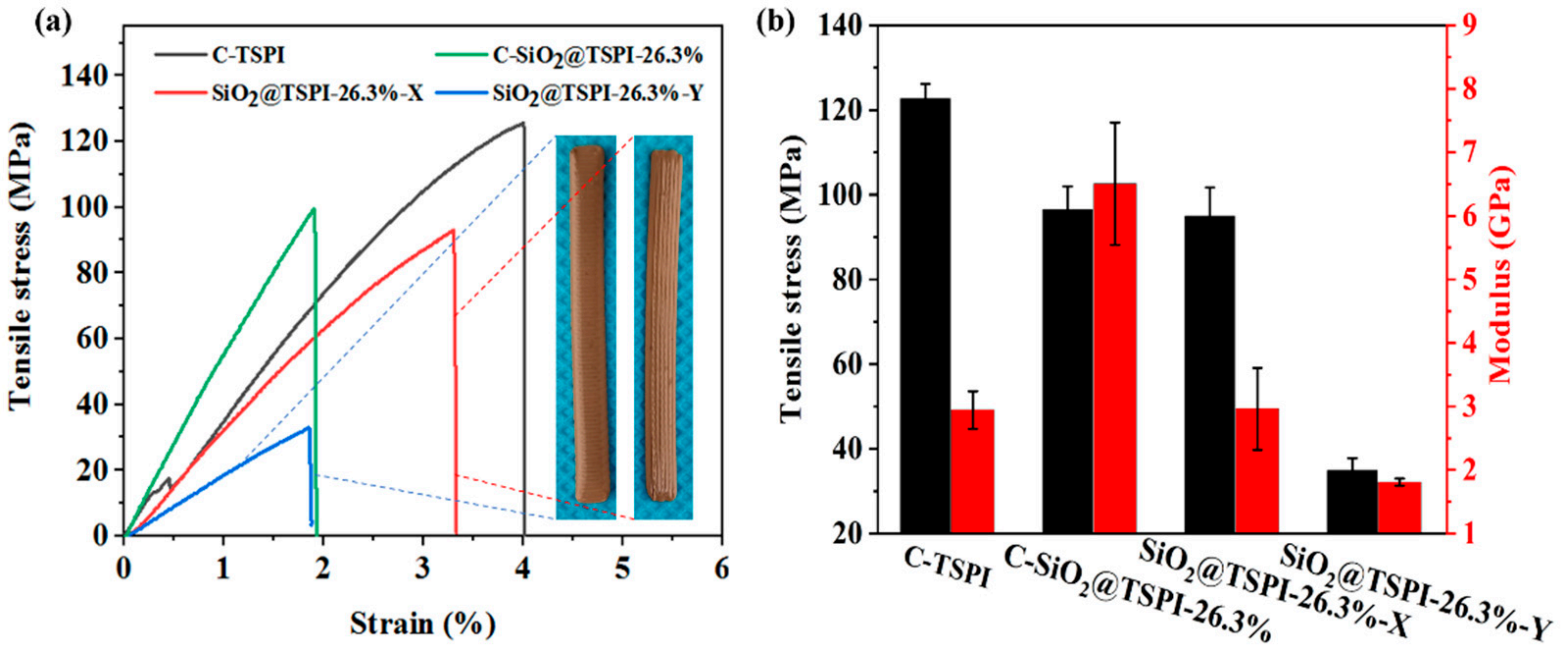
(**a**,**b**): The mechanical properties of TSPI film, SiO_2_@TSPI film, and printed SiO_2_@TSPI composites.

Mechanical properties, which are among the most vital properties of a polymer, are assessed by the uniaxial tensile test. In order to evaluate the strength of printed filaments and the adhesive force among layers, the printed routes of the specimens were divided into transverse (X, SiO_2_@TSPI-26.3%-X) and axial (Y, SiO_2_@TSPI-26.3%-Y) orientations, respectively. Therefore, the impacts of orientation on the printed specimens were analyzed. Meanwhile, the mechanical properties of the plain C-TSPI and C-SiO_2_@TSPI-26.3% films with a lack of orientation were also measured. The results are shown in Figure 10 and Table 3. Compared with the plain C-TSPI films, the C-SiO_2_@TSPI-26.3% films have a decreased tensile strength and increased elastic modulus, which may be due to the aggregation of nano-SiO_2_. In addition, when comparing the C-SiO_2_@TSPI-26.3% films with the SiO_2_@TSPI-26.3%-X composite obtained by printing with the transverse orientation method, there was no significant difference in the average tensile strength. For the C- SiO_2_@TSPI-26.3% films and the print roads of the transverse and axial SiO_2_@TSPI-26.3% composites, the average tensile strengths are 96.46 MPa, 94.97 MPa and 34.89 MPa, respectively, and their average elastic modulus values are 2.96 GPa, 6.51 GPa, and 1.8 GPa, respectively. The decrease in modulus for the printed SiO_2_@TSPI-26.3% composite was caused by a defective stack among the filaments. Furthermore, these results indicate that both the elastic modulus and tensile strength for the Y orientation were related to poor performance. Compared to samples with a printing road in the Y orientation, samples with a printing road in the X orientation showed higher tensile strength and exhibited excellent mechanical properties. Therefore, the printed samples with the X and Y orientations exhibited highly anisotropic behavior [33]. The major reasons for this phenomenon are the limitations of a direct ink 3D printing machine and the poor adhesive force of each layer caused by the poor diffusion and entanglement between the filaments. As a result, a poorer adhesive force between the filaments than between other regions brought about a decrease in the mechanical performance of the whole part.

The 5% weight loss temperature, dimensional shrinkage, and mechanical strength in this work and some published results are listed in Table 4. The SiO_2_@TSPI composites obtained in this work exhibit the lowest shrinkage while maintaining competitive thermal and mechanical properties in comparison with the reported 3D printed composites. The critical factor of MPLA, SLA, UV-assisted DIW, and DLP is using UV-induced free radical polymerization of photosensitive groups in molecular chains to form a crosslinked network. The heat treatment was carried out for thermal imidization or further crosslinking, and, then, high-precision structures were obtained. The introduction of photosensitive agents and other photocurable agents decreased the thermo-mechanical properties compared with the imidization products, and a large dimensional shrinkage occurred. For FDM, the oligomers with phenylethynyl-terminated PI were first prepared. During the process of extrusion at a high temperature, the crosslinked network was formed. Therefore, the manufacturing process is not easy due to the block of inks in the nozzle.

## 4. Conclusions

In summary, we established a simple strategy to 3D print thermal setting polyimides using DIW technology. Based on phenylethynyl end-capped SiO_2_@oligoimide self-supporting ink, the printing parameters of extruding pressure, the moving speed of the nozzle, and z-spacing were studied, and structures with tailored geometry were successfully printed. After the curing procedure was operated, the maximum dimensional shrinkage was only 16%. Additionally, the printed samples exhibited highly anisotropic behavior, which could be described as the average tensile strengths for when the X and Y orientations were 95.0 MPa and 34.9 MPa, respectively. Both the printed SiO_2_@TSPI composite and TSPI showed considerable thermal properties and thermo-stability, and their T5% values were higher than 515 °C. The thermal properties and thermo-stability of the printed SiO_2_@TSPI composite were superior to those of the TSPI composite. Overall, this method may extend the applications of thermosetting polymers by providing a simple and convenient strategy to manufacture 3D parts with outstanding performance.

## Figures and Tables

**Table 1:** Composition, M_n_, and designation of polyamic acids and oligoimides.

Composition (Mole Ratio)	Designation of Polyamic Acids	Designation of Oligoimides	M_n_ (g/mol) Calculated	M_n_ (g/mol) Measured	PDI
PEPA/ODA/6FDA 2/24.47/23.57	PAA	PI	15,000	18,700	1.38
PEPA/ODA/6FDA 2/8.13/7.13	PAA-1	PI-1	5000	5100	1.97

**Table 2:** Data of thermal performance.

Samples	T_g_ (°C)	T_5%_ (°C)	T_10%_ (°C)	Char Yield (%)
C-TSPI-Air	-	519.0	536.6	0.00
C-TSPI-NSi_2_	306.1	525.5	544.5	54.7
SiO_2_@TSPI-26.3%-Air	-	525.9	545.6	24.2
SiO_2_@TSPI-26.3%-NSi_2_	300.7	529.1	546.5	66.0

**Table 3:** Data of mechanical properties.

Sample	Tensile Strength (MPa)	Elastic Modulus (GPa)
C-TSPI	118.95–125.60	2.60–3.15
C- SiO_2_@TSPI-26.3%	90.10–99.67	5.66–7.55
SiO_2_@TSPI-26.3%-X	89.36–102.56	2.29–3.58
SiO_2_@TSPI-26.3%-Y	32.96–38.22	1.75–1.87

**Table 4:** Properties of the reported 3D printed TSPI and that produced in this work.

Printing Method	Structure	T_5%_ (°C) ^a^	Tensile Stress (MPa)	Dimensional Shrinkage (%)	Ref.
MPSL	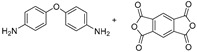	590	80	52	[27]
SLA	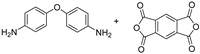	498	---	48	[34]
UV-DIW	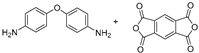	534	---	45	[28]
DLP	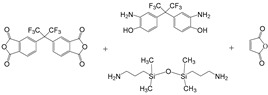	420	24.9	---	[29]
FDM	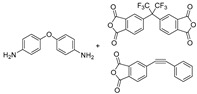	----	86 ± 11	---	[33]
DIW (this work)	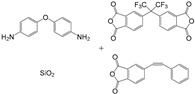	529	95	16	---

^a^ measured in N_2_ atmosphere.

## Data Availability

The data presented in this study are available on request from the corresponding authors.
